# Frost Damage in Tight Sandstone: Experimental Evaluation and Interpretation of Damage Mechanisms

**DOI:** 10.3390/ma13204617

**Published:** 2020-10-16

**Authors:** Shun Ding, Hailiang Jia, Fan Zi, Yuanhong Dong, Yuan Yao

**Affiliations:** 1 College of Architecture and Civil Engineering, Xi’an University of Science and Technology, Xi’an 710054, China; dingshun@stu.xust.edu.cn; 2 Xi’an Engineering Investigation and Design Research Institute of China National Non-Ferrous Metals Industry, Xi’an 710054, China; zifan319@stu.xust.edu.cn; 3 State Key Laboratory of Road Engineering Safety and Health in Cold and High-Altitude Regions, CCCC First Highway Consultants Co., Ltd., Xi’an 710065, China; dyhvic@gmail.com; 4 Urumqi Institute of Central Asia Earthquake, China Earthquake Administration, Urumqi 830011, China; yy8096658@126.com

**Keywords:** building stone, tight sandstones, frost damage, NMR, strength decay, pore structure evolution

## Abstract

Low-porosity tight rocks are widely used as building and engineering materials. The freeze–thaw cycle is a common weathering effect that damages building materials in cold climates. Tight rocks are generally supposed to be highly frost-resistant; thus, studies on frost damage in tight sandstone are rare. In this study, we investigated the deterioration in mechanical properties and changes in P-wave velocity with freeze–thaw cycles in a tight sandstone. We also studied changes to its pore structure using nuclear magnetic resonance (NMR) technology. The results demonstrate that, with increasing freeze–thaw cycles, (1) the mechanical strength (uniaxial compressive, tensile, shear strengths) exhibits a similar decreasing trend, while (2) the P-wave velocity and total pore volume do not obviously increase or decrease. (3) Nanopores account for >70% of the pores in tight sandstone but do not change greatly with freeze–thaw cycles; however, the micropore volume has a continuously increasing trend that corresponds to the decay in mechanical properties. We calculated the pressure-dependent freezing points in pores of different diameters, finding that water in nanopores (diameter <5.9 nm) remains unfrozen at –20 °C, and micropores >5.9 nm control the evolution of frost damage in tight sandstone. We suggest that pore ice grows from larger pores into smaller ones, generating excess pressure that causes frost damage in micropores and then nanopores, which is manifested in the decrease in mechanical properties.

## 1. Introduction

In cold regions, freeze–thaw (hereinafter F-T) action is one of the key processes leading to the deterioration of the physical and mechanical properties of rocks [1,2,3]. Tight rocks with low porosity are widely used as building materials in structures such as foundations, walls and roadbeds [4,5,6,7,8,9,10]. It is generally believed that tight rocks have the advantages of high strength and good weathering resistance. In the past, frost resistance was generally tested in rocks with high porosity [11,12,13], while tight rocks were considered to have good frost resistance [14,15,16]. However, in near-water environments, tight rocks, especially porous sedimentary rocks, are frequently saturated with water, providing sufficient conditions for the development of frost damage, so their resistance to frost action is actually doubtful and warrants systematic study. Due to the rarity of studies on the frost resistance of tight sandstone, the potential frost damage mechanisms in them remain unclear.

Frost damage in rocks is generally characterized in terms of physical and mechanical properties, with indicators such as strength, elastic modulus, porosity, P-wave velocity and pore structure being widely used. Previous studies have pointed out that repeated frost action will lead to significant declines in the mechanical properties of rocks and soils [17,18,19,20]. Bayram [21] conducted F-T experiments on nine types of limestone to predict its uniaxial compressive strength and elastic modulus attenuation, thereby evaluating rock damage in relation to number of F-T cycles. Altindag [22] conducted uniaxial compression tests, Brazilian split tests, point load tests and P-wave velocity tests of rocks undergoing F-T cycles, finding that each index decreased as the number of F-T cycles increased. By studying rocks of various porosities, it was found that the effective porosity increases during the F-T cycle. Rocks with higher initial porosity have faster growth rates and greater increases in porosity [23,24,25]. Attenuation of P-wave velocity in different types of rocks during F-T cycles has been widely accepted. The reason should be that repeated frost action extends internal micro-cracks lengthening the wave propagation path [26,27]. Several scholars have discussed the evolution in the pore structure of rocks with F-T cycles using nuclear magnetic resonance (hereinafter NMR) technology. They found that repeated F-T cycles directly destroy the internal pore structure of the rock, causing the increase in porosity [28,29,30,31]. Moreover, X-ray computed tomography (CT) and scanning electron microscopy (SEM) observations have also confirmed remarkable changes in the microscopic pore structure of rock due to F-T action, clearly showing that micro-cracks inside the rock expand and grow significantly [32,33,34].

Among all the above indexes of frost damage in rocks, P-wave velocity is one of the most popular ones since its test is fast and non-destructive. The feasibility of this method is based on the sensitivity of the propagation of ultrasonic wave to changes in the rock skeleton; it is doubtful whether P-wave velocity can detect frost damage in tight rocks, considering their extremely low porosities. For tight rocks used as building materials, strength and durability are the core properties. Deterioration in rock strength under F-T action is closely related to changes in pore structure. Therefore, investigating the frost damage mechanism in tight sandstone should focus on the evolution of its pore structure. The NMR technology is a promising method in evaluating the pore structure evolution in tight rocks due to its wide measuring range of pore size (from nanopores to macropores). 

In this study, we studied the deterioration in the mechanical properties of tight sandstones and changes in P-wave velocity during 75 F-T cycles (+20 to −20 °C). We used NMR technology to study the pore structure characteristics and their evolution under repeated F-T action. The mechanical strength of tight sandstone during the F-T process is analyzed in terms of decays in tensile, shear and compressive strength and evolution in pores of different types. The feasibility of P-wave velocity serving as the index of frost damage in tight sandstone is verified. Finally, the characteristics and mechanism of frost damage accumulation are discussed.

## 2. Methodology

### 2.1. Sample Preparation

**Figure 1:**
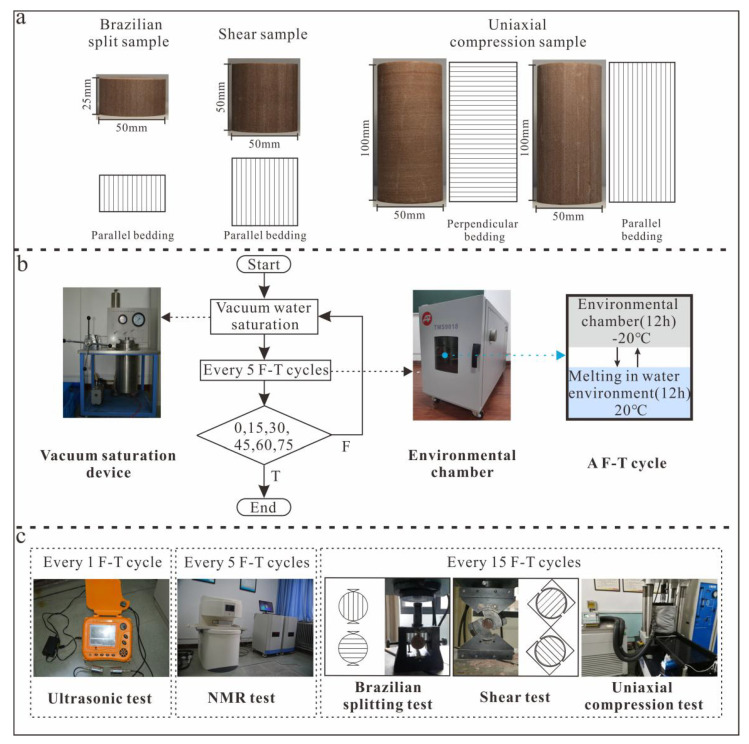
(**a**) Sampling and type of the samples, (**b**) Freeze-thaw cycle test and vacuum saturation, (**c**) the tests of ultrasonic, NMR test, Brazilian splitting, shear, uniaxial compression.

Experimental samples were obtained from Baishui County, Shaanxi Province, China and were fine-grained tight sandstone with reddish-brown bedding. Fresh, similar rock blocks were selected from the same sampling site to ensure consistency. In accordance with the international rock test sample standard, cylindrical samples (diameter = 50 mm) were drilled from rock blocks in the laboratory in both perpendicular and parallel directions to the bedding direction (labeled as S_per_ and S_par_). After cutting and polishing, three groups of cylindrical samples were prepared, with length–diameter ratios of 0.5 (height = 25 mm, used for Brazil split tests), 1 (height = 50 mm, used for variable angle shear tests) and 2 (height = 100 mm, used for uniaxial compression strength tests, NMR tests and ultrasonic tests; Figure 1a).

Samples were placed in an oven at 105 °C for 24 h to ensure dryness. After cooling the rock samples to room temperature in a dry environment, the quality and P-wave velocity were measured. All cylindrical samples were screened in terms of P-wave velocity, density and porosity so that similar samples could be matched. As a result, 12, 48 and 12 S_per_ cylinders, and the same amount of S_par_ cylinders with length–diameter ratios of 0.5, 1 and 2, were selected as the samples. Table 1 shows the mineral particle compositions of the samples according to XRD and their basic physical parameters. Figure 1 shows the experimental samples, procedures and instruments used in this study.

### 2.2. Experimental Design

#### 2.2.1. Freeze–Thaw Cycle Experiments

The selected six groups of samples were sequentially set to undergo 0, 15, 30, 45, 60 and 75 F-T cycles from −20 to +20 °C. All samples were saturated using the vacuum saturation method (pumping to −0.1 MPa for 4 h, soaking in distilled water for more than 48 h) before the test. One F-T cycle consists of 12-h freezing in an environmental chamber at −20 °C and 12-h thawing in water at 20 °C. Samples were re-saturated by the vacuum method every 5 F-T cycles (Figure 1b). Samples were wrapped with plastic film during freezing to prevent water loss.

#### 2.2.2. Mechanical Tests

1. Brazilian split tests

Samples with a length–diameter ratio of 0.5 were used in the Brazilian split tests. The samples were tested after 0, 15, 30, 45, 60 and 75 F-T cycles using an RFP-09 Intelligent Dynamometer (Figure 1c). The rate of axial loading was consistent (0.02 kN/s) for all samples.

2. Varying-angle hear tests

Samples with a length–diameter ratio of 1 were used in the varying-angle shear tests. The samples were tested after 0, 15, 30, 45, 60 and 75 F-T cycles using the RFP-09 Intelligent Dynamometer (Figure 1c). Four shear angles were used: 45°, 55°, 65° and 75°. The loading rates were identical and constant at 0.5 kN/s.

The varying-angle shear test system includes the intelligent test pressure machine and varying-angle shear device. The test principle is that the variable-angle shear device can decompose the applied vertical pressure (*P*) into the shear pressure (*P cos α*) applied on the sample along the shear plane and the normal pressure (*P sin α*) perpendicular to the shear plane. The shear stress and corresponding normal stress at failure on the shear plane can be calculated by Equations (1) and (2):

(1) $$\tau =\frac{P}{bl}cos\alpha$$

(2) $$\sigma =\frac{P}{bl}sin\alpha$$

where *τ* and *σ* are the average shear stress and normal stress of the shear plane, respectively (MPa); *α* is the shear angle, which is the angle between the shear plane and the loading direction (°); *P* is the vertical load at failure (N); and *b* and *l* are the width and length of the shear plane upon failure, respectively (m). Using the Mohr–Coulomb criterion, the cohesion *c* and internal friction angle *φ* were calculated from a linear fitting of the (*σ*, *τ*) plot in the *σ*-*τ* coordinates.

3. Uniaxial compression tests

Samples with a length–diameter ratio of 2 were used in the uniaxial compression tests. The samples were tested after 0, 15, 30, 45, 60 and 75 F-T cycles using a GCTS RTX-1500 system (GCTS Testing Systems, Tempe, AZ, USA; Figure 1c). The rate of axial loading was consistent (0.06 mm/min) for all samples.

#### 2.2.3. Nuclear Magnetic Resonance Tests

By measuring the NMR signal of saturated rocks, the evolution in pore structure of rocks with F-T cycles can be determined. We used an NMR instrument (MacroMR12-150H-I, Suzhou Niumag Analytical Instrument Corporation, Suzhou, China) with a magnetic field strength of 0.3 ± 0.05 T, H proton resonance frequency of 12.77 MHz and coil radio frequency pulse frequency of 1.499 MHz. The major test parameters in this study were SW = 250 W, TE = 0.4 ms, TW = 2000 ms, NECH = 5000 and NS = 16.

Based on the principles of NMR, the *T*_2_ spectrum of pore water in a fully saturated sample directly indicates the total pore volume and pore size distribution [35,36]. There is a corresponding relationship between the *T*_2_ spectrum and the surface area ratio of the pores [37,38]:

(3) $$\frac{1}{T_{2}}=\rho_{2}\frac{S}{V}=\rho_{2}\frac{F_{S}}{r}$$

where $T_{2}$ is the transverse relaxation time; $\rho_{2}$ is the transverse relaxation strength (for sandstone, $\rho_{2}=5\mu m/s$); S/V is the pore face ratio; $F_{S}$ is the pore shape factor (rock pore structures can be considered as a tubular model where $F_{S}=2$); and *r* is the pore radius. The *T*_2_ spectrum distribution of a saturated sample can be transformed into a pore size distribution curve based on Equation (3). In this study the *T*_2_ spectra were obtained for saturated samples with aspect ratios of 2 after every 5 F-T cycles. Before the NMR test, saturated samples were wiped with a moist towel to remove surface water, then immediately wrapped in plastic film to prevent water loss during the test.

#### 2.2.4. Ultrasonic Testing

The P-wave velocities of samples were obtained with a non-metal ultrasonic analyzer (NM-4B; transmission pulse width = 0.1–600 μs, sampling period = 0.1 μs, Beijing Koncrete Engineering Detection Technology Company, Beijing, China). The P-wave velocities in the saturated state were measured after samples with aspect ratio = 2 had been thawed after every 5 F-T cycles. To improve sample–header contact, petroleum jelly was used at the interface. 

## 3. Results

### 3.1. Changes in the Mechanical Properties of Tight Sandstone with F-T Cycling

#### 3.1.1. Tensile Strength

**Figure 2:**
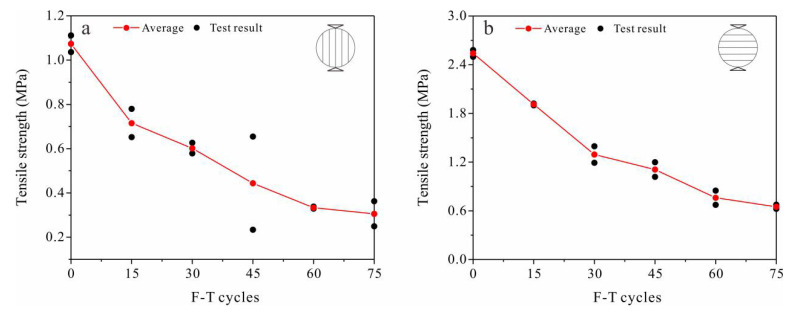
Variations in tensile strength with F-T cycles in sandstone samples with loading directions (**a**) parallel (S_par_) and (**b**) perpendicular (S_per_) to the bedding.

The results of the tensile strength tests of the sandstone samples of two bedding types are shown in Figure 2. With increasing F-T cycles, the tensile strength of tight sandstone was significantly decreased, and the tensile strength of both samples decreased by more than 70% after 75 F-T cycles. This indicates that severe frost damage was caused to the tight sandstone, which is not consistent with the current understanding that tight sandstone has good frost resistance. The tensile strength of the samples decayed rapidly at first (<30 cycles) and then more slowly with further F-T cycles. In addition, the initial tensile strengths of the S_per_ samples were more than twice of the S_par_ samples, besides, the rates of strength reduction of the two types of samples were different. (Figure 2). This reflects the anisotropic characteristics of the tensile strength in bedded sandstones.

#### 3.1.2. Shear Strength Parameters

**Figure 3:**
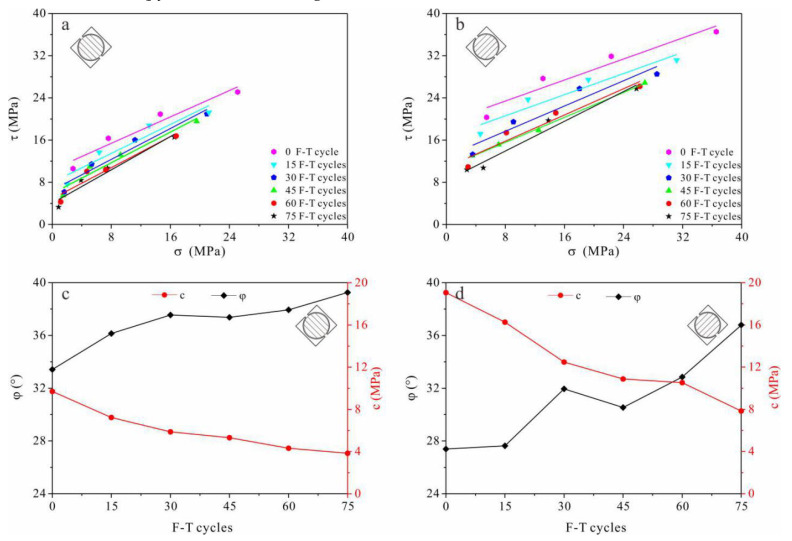
Variation in shear resistance with F-T cycles in sandstone samples with shear planes (**a**,**c**) parallel (S_par_) and (**b**,**d**) perpendicular (S_per_) to the bedding direction.

With increasing F-T cycles, the shear strength of tight sandstone decreased remarkably (Figure 3a,b). The internal friction angle of tight sandstone increased gradually with F-T cycles. The friction angles of S_par_ and S_per_ samples increased by 34.30% to 17.49%, respectively, after 75 F-T cycles (Figure 3c,d). While, the cohesive force of S_par_ and S_per_ samples decreased by 58.87% and 60.35%, respectively (Figure 3c,d). The shear strength parameters of the two types of sandstone samples were different, and the rates at which they decreased were also different after a given number of F-T cycles. This also reflects the anisotropy in the shear strength of bedded sandstones. 

#### 3.1.3. Uniaxial Compressive Strength

**Figure 4:**
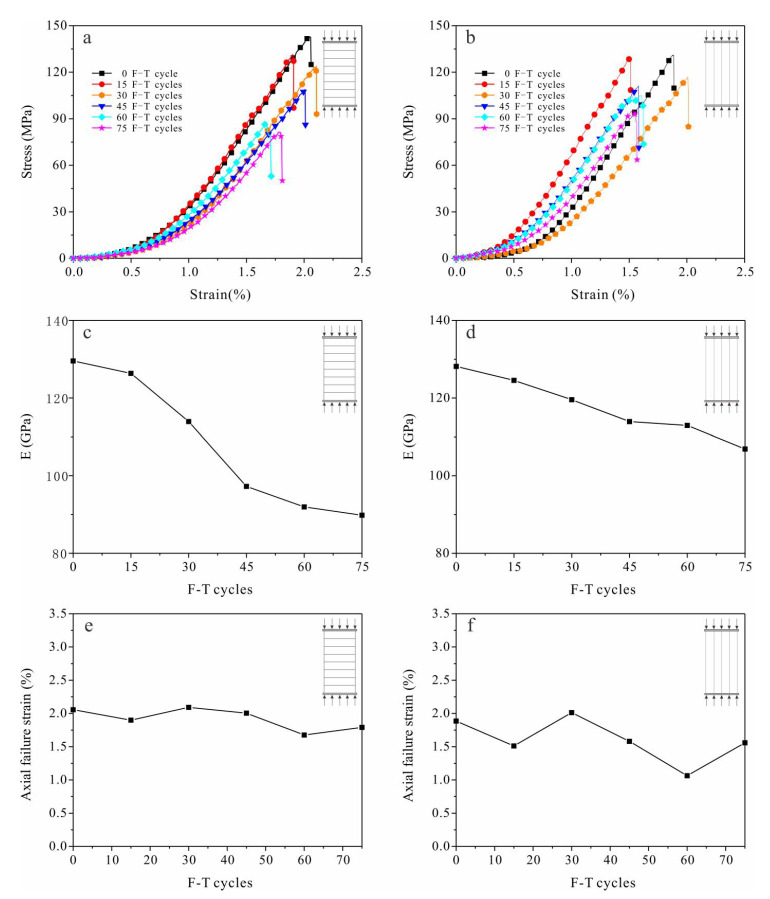
Curves of stress–strain (top), elastic modulus (middle) and axial failure strain (bottom) with F-T cycles of sandstone samples with loading (**a**,**c**,**e**) perpendicular (S_per_) and (**b**,**d**,**f**) parallel (S_par_) to the bedding direction.

All stress–strain curves of sandstone samples went through a three-stage pattern of pore compaction–elastic deformation–stable micro-fracture propagation. Tight sandstone experienced brittle failure under compression indicated by the fact that the axial stress dropped after rupture (Figure 4a,b). Slopes of the elastic-deformation stage decreased gradually with F-T cycles, with the elastic modulus attenuation of S_per_ samples being more obvious. Moduli of the S_per_ and S_par_ samples were reduced by 30.67% and 16.61%, respectively, after 75 F-T cycles (Figure 4c,d). Axial failure strain of both S_per_ and S_par_ samples were decreasing with F-T cycles, and reduced by 0.25% and 0.32% after 75 F-T cycles, respectively (Figure 4e,f).

**Figure 5:**
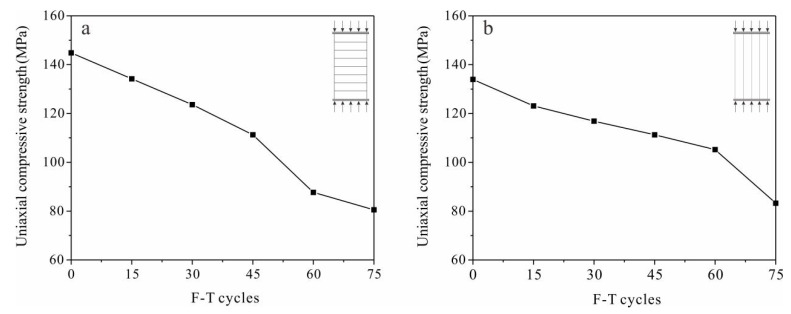
Uniaxial compressive strength with F-T cycles in sandstone loaded (**a**) perpendicular (S_per_) and (**b**) parallel (S_per_) to the bedding direction.

As the number of F-T cycles increased, the uniaxial compressive strength of the sandstone samples gradually decreased (Figure 5). After 75 F-T cycles, the compressive strengths of the S_per_ and S_par_ samples were reduced by 44.39% and 37.85%, respectively.

### 3.2. Changes in Ultrasonic Velocity with F-T Cycles in Tight Sandstone

**Figure 6:**
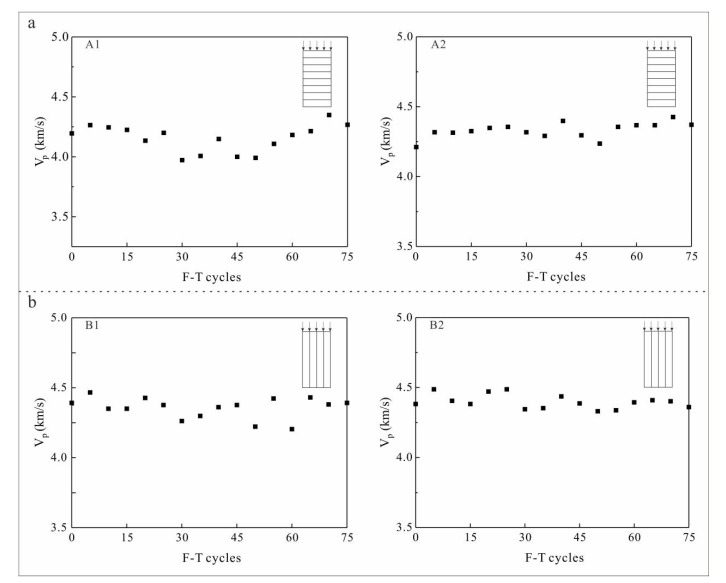
Wave velocity changes with F-T cycles in sandstone samples. Wave velocities propagating (**a**) perpendicular (S_per_) and (**b**) parallel (S_par_) to the bedding plane.

With increases in F-T cycles, the P-wave velocity of sandstone samples fluctuated within a narrow range, without any obvious increasing or decreasing trends (Figure 6). P-wave velocity is frequently adopted to assess the degree of frost damage in rocks. Studies have shown a negative correlation between rock P-wave velocity and the number of F-T cycles [26,27]. However, the results of our experiments indicate that changes in P-wave velocity do not reflect frost damage in tight sandstone. Therefore, it is misleading to use this index to evaluate Frost damage as it may lead to an incorrect conclusion that tight sandstone has good frost resistance.

### 3.3. Changes in Tight Sandstone Pore Structure with F-T Cycles

**Figure 7:**
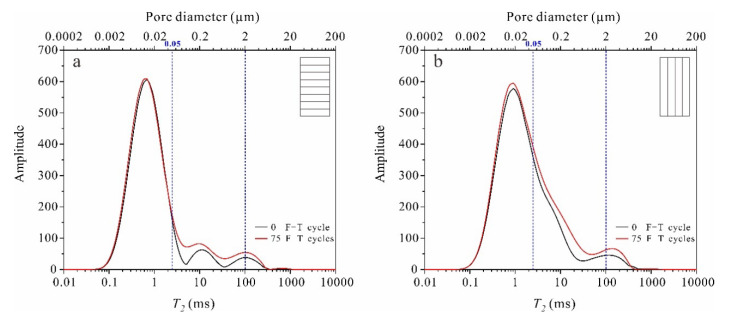
NMR T_2_ spectra and pore size distributions before and after F-T cycles in tight sandstone samples with (**a**) perpendicular bedding (S_per_) and (**b**) parallel bedding (S_par_).

Pores of rocks can be categorized according to diameter. There are several pore classification criteria used in different fields. This paper refers to the classification criteria proposed by Ondrášik et al. based on Kelvin’s equation [39,40]. Pores of >0.1 mm diameter are called macropores, pores with diameters of 2 μm–0.1 mm are mesopores, pores of 50 nm–2 μm are micropores, and pores of <50 nm are nanopores. Figure 7 shows that there were no pores of >0.1 mm diameter in the tight sandstone and most were <50 nm. After 75 F-T cycles, the pore structure changes were mostly in the micropore interval; these pores increased noticeably in volume. Nanopores, which dominated the rock body, did not change obviously.

**Figure 8:**
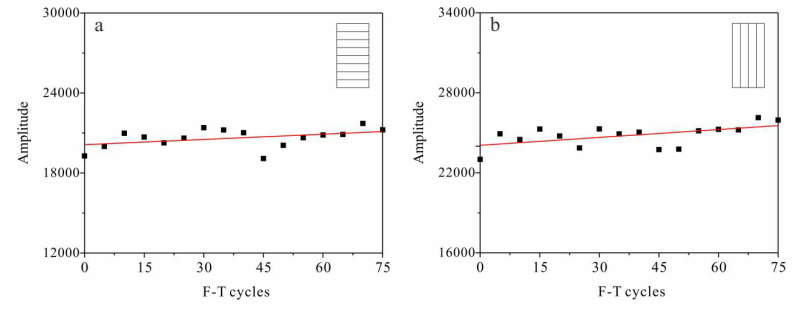
Variation in total *T*_2_ spectral area with F-T cycles in samples with (**a**) perpendicular bedding (S_per_) and (**b**) parallel bedding (S_par_).

There is a linear relationship between a sample’s *T*_2_ spectral area and its porosity. Therefore, variation in *T*_2_ spectral area can be used as an indicator of porosity change. Figure 8 shows variations in total *T*_2_ spectral areas with F-T cycles. The overall trend is slightly upward, with pronounced fluctuation. This indicates that for tight sandstone, changes in the total area of the *T*_2_ spectrum (that is, porosity) cannot effectively reflect the degree of Frost damage.

**Figure 9:**
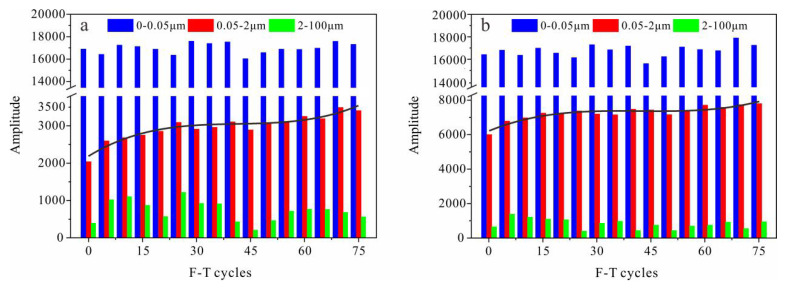
Changes in the total area of aperture-type *T*_2_ spectra with F-T cycles in samples with (**a**) perpendicular bedding (S_per_) and (**b**) parallel bedding (S_par_).

Figure 9 shows the variation in *T*_2_ spectral area with F-T cycles in each pore size category. Neither mesopores nor nanopores showed obvious increases or decreases, while micropores showed a clear upward trend with F-T cycles. The micropores of perpendicular and parallel bedding samples increased by 67% and 35% after 75 F-T cycles, respectively, which values are close to their attenuation ratios of strength. 

## 4. Discussion

### 4.1. Damage Process in Tight Sandstone under F-T Cycles

Strength attenuation and deterioration of the pore structure are direct indicators of rock damage, while indirect indicators such as ultrasonic velocity can also reflect damage. The accumulation of frost damage in rocks can be analyzed based on changes in the above indicators. Experimental results show that after repeated F-T cycles, the tensile strength, shear strength and uniaxial compressive strength in tight sandstone samples with perpendicular and parallel bedding have similar decreasing trends (Figure 2, Figure 3 and Figure 5), while the volume of micropores increase continuously (Figure 9). Changes in the pore structure of tight sandstone demonstrate frost damage accumulation, with similar trends to the attenuations of strength. Therefore, the mechanical strength of tight sandstone is primarily affected by changes to micropores.

P-wave velocity has been widely used to evaluate frost damage in porous rock and soil [26,27]. Measurement of the P-wave velocity of tight sandstone subjected to increasing F-T cycles indicates that it always fluctuates around the initial value without any obvious decreasing trend, suggesting that P-wave velocity is unsuitable for evaluating frost damage in tight sandstone.

### 4.2. Quantifying Pore Water Freezing in Tight Sandstone

The frost damage to porous sandstone is in essence the result of deterioration in pore structure driven by frost-heave pressure. During a typical diurnal F-T cycle, the frost-heave pressure in the pores is induced by water–ice phase transition [15]. Therefore, quantifying the amount of pore water that freezes at a certain temperature is the premise of revealing the damage mechanisms. 

The freezing point of pore water is negatively correlated with pore size; in other words, smaller pores will have a lower freezing point. The pressure difference at the ice–water interface in pores can be calculated by the Young–Laplace equation [41]. This equation describes the pressure difference between the inside and outside of the curved surface of an object and has the following form:

(4) $$\Delta P=2\varsigma H=\varsigma \left(\frac{1}{R_{1}}+\frac{1}{R_{2}}\right)$$

Among them, ΔP is the pressure difference between the inside and outside of the curved surface, also known as the Laplace force; ς is the surface tension of the material, which is equal to the specific surface energy for isotropic materials; *H* is the average curvature of the curved surface; $R_{1}$ and $R_{2}$ are the principal curvature half-means of the curved surface, which are equal for a spherical surface. If the outside of the curved surface contacts other liquids, ς is the interface energy between the two, which is denoted as $\varsigma_{ab}$, where *a* and *b* denote the two substances. In this study, the two substances were water and ice, and the interface between the two can be denoted as $\varsigma_{iw}$. Assuming that a pore is cylindrical with radius $r_{p}$, the curvature of the ice–water interface is $H_{iw}=-\cos\theta /r_{p}$, then the pressure difference between the water and ice is:

(5) $$\Delta P_{iw}{=P}_{i}{-P}_{w}=2\varsigma_{iw}H_{iw}=\frac{-2\varsigma_{iw}cos\theta }{r_{p}}$$

Combined with Equation (4), the relationship between pore radius and the pore water freezing point can be obtained:

(6) $$T_{d}=T_{o}exp\left(\frac{2v_{i}\varsigma_{iw}cos\theta }{Lr_{p}}\right)$$

The freezing temperature of water in pores with different diameters can be calculated through the above equation using the parameter values shown in Table 2 [42].

According to Equation (6) and combined with the distribution characteristics of rock pore size (Figure 7), the unfrozen water content of saturated rock at a certain freezing temperature can be estimated. According to Equation (6), water will not freeze at −20 °C in pores with diameters <5.9 nm, which accounts for around 24% of the total pores. Considering that the pore water contained a certain amount of ions, the ion concentration may also depress the freezing point of pore water [43]. Therefore, the true proportion of unfrozen water should be higher than 24%.

### 4.3. Frost Damage Mechanisms in Tight Sandstone

**Figure 10:**
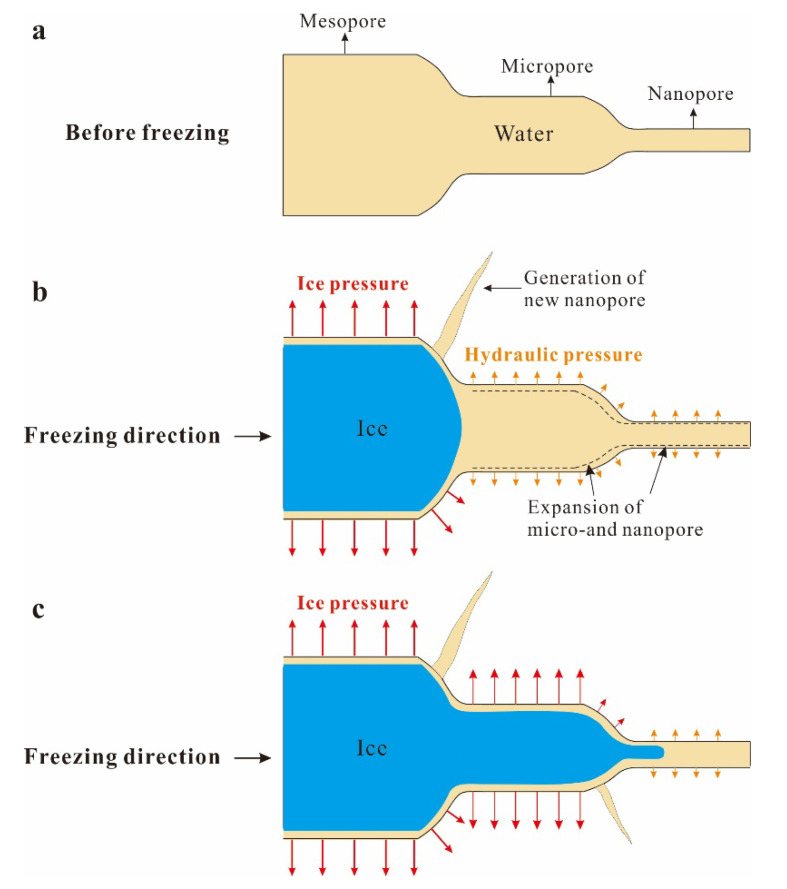
Connected pores in tight sandstone and mechanisms of frost damage (not to scale), (**a**) initial pores filled with water before freezing, (**b**) freezing of pore water initiates from mesopores and (**c**) ice crystal grows into micro- and nanopores.

The experimental results show that the pore structure of frost-damaged tight sandstone exhibits a clear increase in the volume of micropores (Figure 9). It should be the result of sequential freezing of pore water. As temperature decreases in tight sandstone, the pore water in mesopores freezes first, followed by that in micropores and, finally, the water in nanopores. Considering that these pores are connected, the ice crystals in large pores should grow into small ones. Everett [44] proposed a frost damage theory based on the thermodynamic balance of pore water and ice: the excess pressure caused by ice in large pores entering small pores is the cause of frost damage in porous materials. With the continuous growth of ice crystals, frost damage occurs in micropores and nanopores successively (Figure 10). With reference to the negative correlation between the freezing point of pore water and pore size (Equation (6)), we can simply conclude that the level of frost damage in tight sandstone is primarily determined by freezing temperature. 

We take the perpendicular bedding sandstone as an example. Nanopores with diameters <5.9 nm accounted for around 24% of the total pores, and the internal water did not freeze. Therefore, pores >5.9 nm control the evolution of frost damage in tight sandstone. Under F-T cycles, damage to pores will produce new nanopores or cause them to expand into micropores (Figure 10b). The fluctuation in the volume of nanopores with F-T cycle is the superposition of generation of new nanopores and expansion of the existing ones (Figure 10b). While, the increasing volume of micropores with continuous F-T cycles is the combined result of frost-driven expansion of nanopores (both the newly born and the existing) and micropores (Figure 10c). 

The damage evolution process can be explained from the perspective of fracture mechanics. The pore expansion (or crack propagation) rate is controlled by the stress intensity factor, which is positively correlated to both pore length and frost-heave pressure [45]. The magnitude of frost-heave pressure is determined primarily by volumetric expansion, which is restricted by the pores. Smaller pores usually have greater stiffness and, thus, provide greater restriction [46]. At first, nanopores and micropores expanded under repeated F-T cycles and pore length increased but, theoretically, the frost-heave pressure should have decreased. Consequently, pore expansion slowed down or eventually ceased; frost damage was predominately caused by the generation of new pores. With the expansion of these newly generated pores, frost damage accumulated rapidly again [47,48]. Within the above regime of frost damage evolution, mechanical properties decay continuously (Figure 2, Figure 3, Figure 4 and Figure 5). 

Based on the above experimental results and analysis, we consider that frost damage increases the volume of micropores, which is the direct cause of decreases in the strength of tight sandstone. Change in the P-wave velocity of tight sandstone is not sensitive to increases in the number of micropores, so it is unsuitable for evaluating the degree of frost damage in tight sandstone.

## 5. Conclusions

Contrary to the tenet that tight rocks have good frost resistance, our results indicate that tight porous rocks in near-water environments may experience severe decay in mechanical properties due to F-T cycles. In this paper, trends in tensile strength, uniaxial compressive strength, shear strength and P-wave velocity with F-T cycle were studied in two types of tight bedded sandstone. Variation in the internal pore structure of sandstone was tested by NMR technology. The process of damage to the tight sandstone pore structure was discussed and the following conclusions were obtained.

(1) The mechanical strength of tight sandstone exhibited a decreasing trend with increasing F-T cycles. After 75 cycles, the tensile strength decreased by >70%, the cohesive force decreased by around 60%, and the uniaxial compressive strength decreased by around 40%.

(2) With F-T cycle increasing, the P-wave velocity of sandstone samples fluctuated within a small range, without any obvious increase or decrease. Therefore, changes in P-wave velocity cannot quantify frost damage in tight sandstone. Using it as the index of frost damage in tight rocks is misleading and may lead to an erroneous conclusion that tight sandstone has good frost resistance.

(3) The pores of tight sandstone can be divided into nanopores, micropores and mesopores. Nanopores account for >70% of the pores. Under F-T cycling, the total pore volume and nanopore and mesopore contents do not change significantly, while the micropore volume shows a continuously increasing trend. 

(4) At a temperature of −20 °C, a certain amount of unfrozen water remains inside tight sandstone, mainly in nanopores with pore diameters <5.9 nm. Because of the amounts of ions in pore water, the true proportion of unfrozen water should be higher than 24%. As temperature decreases, ice crystals grow into the pores sequentially from large to small; hence, frost damage occurs in micropores and then nanopores. The level of frost damage in tight sandstone is primarily determined by freezing temperature.

(5) The fluctuation in the volume of nanopores with F-T cycle is the superposition of generation of new nanopores and the expansion of the existing ones. While, the increasing volume of micropores with continuous F-T cycles is the combined result of frost-driven expansion of nanopores (both the newly born and the existing) and micropores.

## Figures and Tables

**Table 1:** Mineral composition and physical properties of the samples.

Petrology (%)				Porosity (%)	Dry Density (g/cm^3^)	P-Wave Velocity (km/s)
Calcite	Quartz	Albite	Dolomite			
1.6	75.8	6.1	16.5	3.37	2.60	4.41

**Table 2:** Parameters for calculating pore size and pore water freezing point.

*v_i_* (m³/kg)	*ζ_iw_* (N/m)	*L* (J/kg)	*T_o_* (K)	Cos *θ*
0.001091	0.032	3.34 × 10^5^	273.16	−1
